# Genetic and Environmental Risk for Chronic Pain and the Contribution of Risk Variants for Major Depressive Disorder: A Family-Based Mixed-Model Analysis

**DOI:** 10.1371/journal.pmed.1002090

**Published:** 2016-08-16

**Authors:** Andrew M. McIntosh, Lynsey S. Hall, Yanni Zeng, Mark J. Adams, Jude Gibson, Eleanor Wigmore, Saskia P. Hagenaars, Gail Davies, Ana Maria Fernandez-Pujals, Archie I. Campbell, Toni-Kim Clarke, Caroline Hayward, Chris S. Haley, David J. Porteous, Ian J. Deary, Daniel J. Smith, Barbara I. Nicholl, David A. Hinds, Amy V. Jones, Serena Scollen, Weihua Meng, Blair H. Smith, Lynne J. Hocking

**Affiliations:** 1 Division of Psychiatry, University of Edinburgh, Royal Edinburgh Hospital, Edinburgh, United Kingdom; 2 Centre for Cognitive Ageing and Cognitive Epidemiology, University of Edinburgh, Edinburgh, United Kingdom; 3 Department of Psychology, University of Edinburgh, Edinburgh, United Kingdom; 4 Institute for Genetics and Molecular Medicine, University of Edinburgh, Western General Hospital, Edinburgh, United Kingdom; 5 Institute of Health and Wellbeing, University of Glasgow, Glasgow, United Kingdom; 6 23andMe Inc., Mountain View, California, United States of America; 7 Pfizer WRD, Human Genetics and Computational Biomedicine, Granta Park, Cambridge, United Kingdom; 8 Division of Population Health Sciences, University of Dundee, Ninewells Hospital and Medical School, Dundee, United Kingdom; 9 The Institute of Medical Sciences, University of Aberdeen, Foresterhill, Aberdeen, United Kingdom; Children’s Hospital Boston and Harvard Medical School, UNITED STATES

## Abstract

**Background:**

Chronic pain is highly prevalent and a significant source of disability, yet its genetic and environmental risk factors are poorly understood. Its relationship with major depressive disorder (MDD) is of particular importance. We sought to test the contribution of genetic factors and shared and unique environment to risk of chronic pain and its correlation with MDD in Generation Scotland: Scottish Family Health Study (GS:SFHS). We then sought to replicate any significant findings in the United Kingdom Biobank study.

**Methods and Findings:**

Using family-based mixed-model analyses, we examined the contribution of genetics and shared family environment to chronic pain by spouse, sibling, and household relationships. These analyses were conducted in GS:SFHS (*n* = 23,960), a family- and population-based study of individuals recruited from the Scottish population through their general practitioners. We then examined and partitioned the correlation between chronic pain and MDD and estimated the contribution of genetic factors and shared environment in GS:SFHS. Finally, we used data from two independent genome-wide association studies to test whether chronic pain has a polygenic architecture and examine whether genomic risk of psychiatric disorder predicted chronic pain and whether genomic risk of chronic pain predicted MDD. These analyses were conducted in GS:SFHS and repeated in UK Biobank, a study of 500,000 from the UK population, of whom 112,151 had genotyping and phenotypic data. Chronic pain is a moderately heritable trait (heritability = 38.4%, 95% CI 33.6% to 43.9%) that is significantly concordant in spouses (variance explained 18.7%, 95% CI 9.5% to 25.1%). Chronic pain is positively correlated with depression (ρ = 0.13, 95% CI 0.11 to 0.15, *p* = 2.72x10^-68^) and shows a tendency to cluster within families for genetic reasons (genetic correlation = 0.51, 95%CI 0.40 to 0.62, *p* = 8.24x10^-19^). Polygenic risk profiles for pain, generated using independent GWAS data, were associated with chronic pain in both GS:SFHS (maximum β = 6.18x10^-2^, 95% CI 2.84 x10^-2^ to 9.35 x10^-2,^ *p* = 4.3x10^-4^) and UK Biobank (maximum β = 5.68 x 10^−2^, 95% CI 4.70x10^-2^ to 6.65x10^-2^_,_ *p* < 3x10^-4^). Genomic risk of MDD is also significantly associated with chronic pain in both GS:SFHS (maximum β = 6.62x10^-2^, 95% CI 2.82 x10^-2^ to 9.76 x10^-2^_,_ *p* = 4.3x10^-4^) and UK Biobank (maximum β = 2.56x10^-2^, 95% CI 1.62x10^-2^ to 3.63x10^-2^_,_ *p* < 3x10^-4^). Limitations of the current study include the possibility that spouse effects may be due to assortative mating and the relatively small polygenic risk score effect sizes.

**Conclusions:**

Genetic factors, as well as chronic pain in a partner or spouse, contribute substantially to the risk of chronic pain for an individual. Chronic pain is genetically correlated with MDD, has a polygenic architecture, and is associated with polygenic risk of MDD.

## Abstract 2

Andrew M. McIntosh and colleagues investigate the genetic and environmental factors associated with chronic pain and and major depressive disorder.

## Author Summary

**Why Was This Study Done?:**

- Genetic factors and the environment you share with your nuclear family, siblings, or spouse may determine your risk of chronic pain.
- Depression is also associated with chronic pain, but whether this relationship is explained by shared genetic factors, environment, or both is not known.
- We sought to investigate these issues using genetic data and family environmental information from Generation Scotland: Scottish Family Health Study and UK Biobank.

**What Did the Researchers Do and Find?:**

- Using data from the family-based Generation Scotland study, we found that genetic factors and the environment you share with your partner/spouse are important risk factors for the development of chronic pain.
- Shared genetic and environmental factors also partly explained the association between chronic pain and depression.
- Finally, we found evidence showing that the genetic contribution to chronic pain arises through the combined effect of many different genetic risk factors and that the cumulative effects of genetic risk factors for depression increased an individual’s chance of having chronic pain.

**What Do These Findings Mean?:**

- Both genetic factors and chronic pain in a partner or spouse contribute to the risk of chronic pain for an individual.
- Chronic pain is caused by an accumulation of many small genetic effects and is associated with some of the same genetic and environmental risk factors that confer risk of depression.

## Introduction

Chronic pain and major depressive disorder (MDD) are highly prevalent and frequently comorbid conditions [1] that complicate chronic physical disease and are leading causes of disability globally [2]. The causes of chronic pain and depression are poorly understood, although there is evidence of a contribution from genetic factors in each disorder [3,4]. One means of identifying the magnitude of genetic contributions to a disorder is to use a family-based study design. Family-based studies can be used to determine the heritability of a trait by examining the trait correlations between pairs of relatives of varying proximity.

Chronic pain can be considered as a unified, heritable phenotype. Using a family-based approach involving 7,644 individuals from 2,195 extended families from Generation Scotland: Scottish Family Health Study (GS:SFHS), Hocking et al. demonstrated that severe chronic pain has a heritability of 30% [5]. In twin studies, the broad sense heritability of chronic pain has also been estimated similarly at 32% [6]. Williams et al. [7] examined pain reporting at seven sites among the twins and found a single “pain reporting” factor with a heritability of 0.46, which accounted for 95% of the variance of correlation between reporting at different sites. Separately, Vehof et al. [8] identified a latent trait with a heritability of 0.66 that explained the co-occurrence of a number of different chronic pain syndromes in twins, suggesting a common aetiological pathway with a strong genetic basis. The genetic architecture of chronic pain is, however, unknown, and while human and animal studies suggest potentially a polygenic contribution of many common variants to differences in liability [9], to our knowledge, this supposition remains unproven. The heritability of MDD has been estimated, from a meta-analysis of twin studies, at around 37% [10,11], similar to estimates for chronic pain. Whilst the majority of the heritability of MDD is not explained by the risk variants discovered to date, the genetic architecture of MDD is understood to be highly polygenic, with multiple loci of low-penetrance acting additively to confer liability to the disorder [12,13].

The risk factors and associations of chronic pain and depression are also numerous and overlapping [14–21]. Since chronic pain and depression are amongst the most disabling conditions worldwide, it is important to understand the nature of their co-occurrence and whether this is caused by shared genetic or environmental factors. Studies that quantify the relative roles of genetics and shared and unique environment in chronic pain, including those that seek to explain its correlation with depression, are few. One study of 3,266 female twins estimated the cross-trait correlation between depression and pain and found a latent trait that explained the covariance between chronic widespread pain and major depression, with 86% heritability [22]. Another study, among women in the Swedish Twins Registry, examining four “functional somatic syndromes” (including chronic widespread pain (CWP) and MDD), identified two latent traits with quantified additive genetic and non-shared environmental contributions [23]. One of these was mainly genetic and contributed significantly to the variance of both conditions (0.29 for CWP; 0.76 for MDD); the other was mainly environmental and did not contribute to MDD, but did to CWP (0.81). Such studies may help to clarify how these two common conditions are related and could be used to help manage disease risk at the population level.

The main aim of the current study was to assess the genetic and environmental contributions to pain and its comorbidity with MDD. Using data from GS:SFHS [24], we estimated the magnitude of the contribution to chronic pain from genetic factors, and from shared and unique environmental influences using pedigree-based analyses. By examining the effects of shared environment whilst simultaneously modelling genetic factors, we were able to independently assess and separate both sources of phenotypic variance in a single model. Next, we measured the correlation between chronic pain and MDD using bivariate pedigree-based analysis. By modelling genetics and shared environment, we sought to identify and measure the independent contributions of these factors to the observed covariance between chronic pain and MDD. In order to test for a shared genetic architecture for chronic pain and MDD using independent and convergent data, we then conducted a polygenic profile scoring analysis. We used data from two large-scale and independent genome-wide association studies (GWAS) of chronic pain and MDD to prepare a polygenic risk profile for individuals in GS:SFHS and UK Biobank. We then examined whether polygenic risk of MDD was associated with chronic pain using molecular genetic data, and if polygenic risk of chronic pain predicted MDD.

## Methods

Two cohorts were used to examine the genetic and environmental contributions to chronic pain and its relationship with depression: GS:SFHS (*n* = 23,960) and UK Biobank (UKB, *n* = 112,151). GS:SFHS, having a family-based design, was used to estimate the contributions of genetic factors and of shared and unique environment to chronic pain and its correlation with MDD. GS:SFHS includes a number of different genetic relationships, from first degree to more distant relationships beyond second cousin. By examining the correlation in chronic pain between relatives of different proximities, it is possible to estimate trait heritability. However, these estimates can be confounded by the effects of shared environment, and for this reason, we included the effects of these shared environments to adjust these heritability estimates and also assess their independent contribution to phenotypic variance.

GS:SFHS and UKB were then both used to test the genetic relationship between chronic pain and depression risk scores and their associated phenotypes using polygenic risk score analysis. Marker effects were estimated from independent GWAS studies that did not include either the GS:SFHS or UKB study samples. This included the output from a collaborative GWAS analysis between Pfizer and 23andMe.

All GS:SFHS individuals provided written informed consent to participation and for use of their data and samples for medical research. Ethical approval for the study was given by the National Health Service Tayside committee on research ethics (reference 05/s1401/89). UK Biobank (http://www.ukbiobank.ac.uk) received ethical approval from the Research Ethics Committee (reference 11/NW/0382) and the present analyses were conducted under UK Biobank data application numbers 4844 and 3501. All UK Biobank participants provided written informed consent. Summary data from a previous genetic study of chronic pain (conducted in collaboration with Pfizer-23andMe) were also used in the current study. The original genetic study was approved by the Institutional Research Board, and 23andMe received ethical approval from the Ethical and Independent Review Services. All 23andMe participants provided written informed consent.

### Generation Scotland: Scottish Family Health Study

GS:SFHS is a family-structured, population-based cohort recruited at random through general medical practices across Scotland. The protocol for recruitment is described in detail elsewhere [24,25]. The cohort consists of 23,960 individuals over 18 y of age who were recruited if they had at least one other family member willing to participate. Pedigree information was available for all participants, and this has subsequently been validated against estimated relatedness using genome-wide single nucleotide polymorphism (SNP) data on 19,995 individuals. All individuals from GS:SFHS provided data for the current analyses (median age = 47.9, 41% male, 59% female), of whom 19,511 also provided genotype data for risk profiling after quality control (mean age  =  47.50, standard deviation [SD]  =  14.98) (*n* = 11,514 female, *n* = 7,997 male).

Using pedigree information in GS:SFHS, we have created variables to represent the random effect from genetic and environment factors for linear mixed modelling. We created the genetic relationship matrix using all participants as a means of estimating the heritability of specific traits and their genetic correlation between trait pairs. In addition, we fitted several variables in the model to estimate the effects of shared environment. These included measures of common household, common spouse/partner, and a measure representing siblings that shared a common parental and sibling environment. Further details of these measures are provided in S1 Text.

### Assessment of Chronic Pain in Generation Scotland

GS:SFHS participants (*n* = 16955) completed a questionnaire assessment of chronic pain as previously described by Hocking et al. [26]. This included, first, a validated chronic pain identification questionnaire, in which chronic pain was ascertained in the event of positive responses to two questions: (1) Are you currently troubled by pain or discomfort, either all the time or on and off? (2) Have you had this pain or discomfort for more than 3 months? [27,28] Secondly, severity of chronic pain was determined by the Chronic Pain Grade (CPG) questionnaire, a validated seven-item instrument that measures the pain intensity and pain-related disability and grades severity from Grade 1 (low intensity, low disability chronic pain) to Grade 4 (high intensity, high disability, severely limiting chronic pain) [28–30].

### Assessments of MDD in Generation Scotland

All GS:SFHS participants were invited to complete a screening questionnaire for MDD, as described previously [31]. Those screening positive were invited to complete the Structured Clinical Interview for the Diagnostic and Statistical Manual of the American Psychiatric Association (SCID, DSM version 4) and were subsequently diagnosed with MDD if they fulfilled DSM-IV criteria. Individuals who screened negative for MDD or who screened positive but did not fulfil criteria were diagnosed as being free from MDD. Individuals who declined to complete the screening questionnaire or the SCID had their MDD status set to missing. Details of the genotyping and quality control procedures for each cohort are given in the S2 Text.

### UK Biobank

UK Biobank is a health research resource that aims to improve the prevention, diagnosis, and treatment of a wide range of illnesses. Between the years 2006 and 2010, approximately 500,000 people were recruited from across the UK [32]. For the present study, 112,151 community-dwelling individuals (58,914 females, 53,237 males) aged 40 to 73 years (mean = 56.91 years, SD = 7.93) with genome-wide genotyping were available. Individuals were assigned to self-declared MDD if they stated they had previously suffered from clinical depression in the past during their nurse-led assessment (variable no. 20002).

### Assessment of Chronic Pain in UK Biobank

To identify chronic pain participants were asked, “In the last month have you experienced any of the following that interfered with your usual activities?: headache, facial pain, neck or shoulder pain, back pain, stomach or abdominal pain, hip pain, knee pain, pain all over the body.” With each positive response, they were asked whether the pain had lasted for at least 3 mo, and individuals who reported at least one of these pains lasting for at least 3 mo were defined as having “chronic pain.” Chronic pain in UK Biobank was then quantified based on a previous report [1], which categorised chronic pain in ascending order based on whether a single site was affected, two to three sites were affected, or if the pain was widespread and diffuse, affecting four to seven sites or “pain all over the body.” Ninety-three thousand, five hundred and seventy-one individuals provided useable pain data.

### Assessments of MDD in UK Biobank

Individuals with probable MDD in UK Biobank were identified using the definition used by Smith et al. [33]. This definition used information from a touchscreen assessment that included questions from the Patient Health Questionnaire and items of help-seeking for mental health. The final sample of individuals used in the current analysis, who provided both probable MDD and genotyping data, was 27,994 controls and 6,135 with probable major depressive disorder.

### Polygenic Profiling

Polygenic profiling estimates an individual's burden of common risk variants for a given trait by summing trait-associated alleles, weighted by their marker effects, across the genome to create a polygenic risk score (PGRS) within each subject. This process requires GWAS summary statistics from an independent training subset, from which the marker weights (log odds ratio) and a SNP-list ranked by their evidence for association (usually *p*-values) can be obtained.

The training set for chronic pain was designed by Pfizer and conducted in collaboration with 23andMe on their participants. In this Pfizer-23andMe study, validated pain questionnaires, using identical case and severity measures to those used in GS:SFHS, were completed by >32,000 research participants. 23andMe research participants provided written informed consent to take part in this research under a protocol approved by the Ethical and Independent Review Services, an AAHRPP-accredited institutional review board. The number of unrelated Europeans that were included after relatedness removal was 10,780 cases and 12,552 individuals categorised as “no pain” controls. Chronic pain phenotypes for 23andMe participants were defined (as for GS:SFHS). For each pain phenotype, individual SNP effects (odds ratios and 95% CIs) were calculated in PLINK using a linear regression analysis after adjustment for age, sex, body mass index (BMI), current manual labour and previous manual labour and the first five population principal components. Further details of the genotyping and quality control performed by 23andMe are provided in the S2 Text.

Genetic risk of MDD was estimated using published independent GWAS dataset from the Psychiatric Genomics Consortium (PGC) Major Depressive Disorder Working Group. Details of the quality control, imputation, and analysis have been previously provided elsewhere [12].

Chronic pain and MDD polygenic risk profile scores for all individuals in GS:SFHS and UKB with useable GWAS data were then estimated. Polygenic risk profiles were estimated genome-wide using four arbitrarily defined nominal *p*-value thresholds in the original discovery GWAS (*p*-values = 0.01, 0.05, 0.1 and 0.5) producing a score in a second dataset at each corresponding threshold. Statistically significant association of chronic pain in GS:SFHS and UKB using marker weights estimated in Pfizer-23andMe GWAS data was assumed to infer a polygenic risk architecture. Statistically significant association of chronic pain using MDD polygenic profile scores (or vice versa) was assumed to infer an overlapping polygenic risk architecture.

### Statistical Analysis

#### Pedigree-based analysis of chronic pain

Pedigree-based analysis of chronic pain was conducted solely in the GS:SFHS dataset using the Chronic Pain Grade (CPG 0–4) variable. Using a generalised linear mixed-model, we estimated the relative contributions of the random effect from (1) genetic factors (using the additive genetic [or numerator] relationship matrix) calculated from the pedigree, and (2) environmental factors from the parent (using the sib variable), previous/current spouse (using the spouse variable), and household (using the two household variables). The magnitude of each environmental effect (parent, spouse, household) was estimated in a model with the presence of a genetic variance component to reduce confounding of each environmental effect by genetic relatedness. All fixed and random effects were estimated in the MCMCglmm package for Bayesian Generalised linear mixed models [34] and in the R package ASReml-R [35] (version 3) in R version 3.2.2. Since MCMCglmm can adequately model the genetic and environmental contributions to binary and ordinal traits using Markov Chain Monte Carlo sampling, MCMCglmm was used as the primary methodology for estimating the effects sizes for each variance component. Sex, age, and age^2^ were used as fixed effects throughout. We checked for adequate coverage of each model estimate by graphically examining the trace of each parameter. We adjusted for autocorrelation by thinning the sampling frequency using the “thin” option in MCMCglmm, which was set to 50 samples, and we used between 85,000 to 500,000 iterations after a burn-in of 15,000 samples. Each model was run a minimum of three times to check for consistency.

In order to select the best-fitting model to the data, we employed two convergence methodologies, one in each software package. Firstly, we estimated goodness-of-fit in the MCMCglmm package using Deviance Information Criterion (DIC), with larger values indicating poorer fitting models. Secondly, we used ASReml-R to compare goodness-of-fit by estimating the difference in log-likelihood between two models, which was then compared to a chi-squared distribution with the relevant degrees of freedom for the number of parameters that differed between the models. A significant *p*-value indicated a better fitting model. In each case we started with a model containing only a genetic effect, then applied models containing the genetic effects plus one of the three environmental effects (parent, spouse, and household). After choosing the best model from those estimated, we attempted to improve the model fit by adding in a second environmental variable. Where the DIC or log-likelihood could not be further improved, we selected the reduced model as the best fit for the available data and provided the effects from MCMCglmm.

#### Pedigree-based bivariate analyses of pain and MDD

In order to examine the relationship between chronic pain and MDD in GS:SFHS, we used a generalised linear mixed model as implemented in ASReml-R. We estimated the “bivariate” phenotypic correlation between chronic pain and MDD first before providing the genetic correlation coefficient and environmental coefficient for any significant shared environments estimated from the univariate model. The genetic correlation coefficient is an estimate of the correlation between the additive genetic components of chronic pain and MDD. Similarly, the environmental correlation coefficient estimates correlation between the shared environmental components of chronic pain and MDD.

The significance of each correlation was estimated using two nested “null” models. The first null genetic model was constrained to have a genetic correlation of zero, whereas the null environmental model was constrained to have an environmental correlation of zero. Each model was compared to the full (estimated) model and the significance of each effect was determined using the likelihood ratio test (LRT) by comparing two times the difference in the log likelihood to a chi-squared distribution with 1 degree of freedom. Standard errors for each correlation coefficient were also determined using the variance components provided by ASReml-R. In order to account for relatedness and to adjust for the fixed effects of sex, age, and age^2^ (i.e., age squared), the significance of the phenotypic correlation coefficient was estimated using the ratio of its effect size to its standard error in ASReml-R. The significance was then estimated by comparison of this figure to a z-distribution and the calculation of a two-tailed *p*-value.

#### Polygenic profiling

Polygenic profiling of the GS:SFHS sample was conducted in PRSice [36] using the marker weights estimated in the Pfizer-23andMe dataset for the CPG trait in this independent discovery sample. Details of the numbers of SNPs contributing to each polygenic score are given in S2 Table. The polygenic risk scores were first used to test their association with chronic pain in GS:SFHS using the profile score as a fixed effect within a generalised linear mixed model implemented in MCMCglmm. Additionally, sex, age, age^2^, and four multidimensional scaling components were also used as fixed effects. Each model was adjusted to take account of relatedness between subjects using the relationship matrix. The significance of the association between polygenic risk profile scores and chronic pain in GS:SFHS was estimated using the modal value and the 95% credible interval from the posterior density. The variance explained by the polygenic profile score was estimated by multiplying the profile score by its corresponding regression coefficient and estimating its variance [37] in ASReml-R. This value was then divided by the variance of the observed phenotype itself to yield a coefficient of determination between 0 and 1. We then tested for the association between polygenic profile scores for chronic pain and MDD in GS:SFHS.

We then profiled all individuals in GS:SFHS using the publically available PGC dataset for MDD. Polygenic profile scores for MDD were then tested for their association with MDD in GS:SFHS as a means of assessing their validity and to identify a theoretical upper bound for their accuracy in predicting chronic pain. Secondly, we estimated the strength of the association between polygenic profile score for MDD and chronic pain in GS:SFHS using the same models as described in the previous paragraph.

Where a significant association was found between polygenic risk of chronic pain and either chronic pain or MDD in GS:SFHS, we repeated the analysis in UKB using their different chronic pain phenotype which is defined, in contrast to GS:SFHS, on the extent rather than on the intensity of the chronic pain. Where significant associations were found between polygenic risk of MDD and chronic pain in GS:SFHS, these analyses were repeated excluding individuals with major depression (experienced at any point in their life) in order to test whether the same relationship between polygenic risk of MDD and chronic pain was present in the unaffected population.

Associations that were shown to be significant in GS:SFHS were then repeated in UKB using unrelated individuals only. Polygenic risk profile scores were examined for their association with observed phenotypes in ASReml-R using the same methods, but without the inclusion of a GRM due to the large dataset and unrelated nature of the filtered UKB study population used in the current investigation.

## Results

### Univariate Analysis of Chronic Pain Grade in GS:SFHS

Five models were initially estimated for the chronic pain (CPG) dependent variable. In each case the model included a genetic effect, then each of the three environmental effects individually (“sib” [common parent], spouse or household). The model including the additive genetic effect plus that of spouse was a better fit than the model that only included the additive genetic effect alone, either by selection of the model with the lowest Deviance Information Criterion (DIC) or through the likelihood ratio test (LRT). The effect of additive genetic effects and spouse alone was also superior (in terms of DIC) to either of the other two models that included additive genetic effects and “sib” alone, or additive genetic effects and household alone. In a second step, we added further environmental effects to the model that included additive genetics and spouse alone, but we could not improve on the model fit adjudicated using either the DIC or LRT. Each model is reported in S1 Table.

The heritability of chronic pain was substantial, accounting for 38.4% (95% CI 33.6% to 43.9%) of the variation in CPG score. Shared environment with a spouse accounted for approximately 18.7% (95% CI 9.5% to 25.1%) of the variation in susceptibility to chronic pain. The magnitude of this association was approximately half of the variance component explained by additive genetic factors. The residual unexplained variance, reflecting measurement error, poor reliability, and non-shared environment, accounted for just under half of the variance in liability to chronic pain (variance attributed 42.9%).

### Bivariate Analysis of Chronic Pain Grade in GS:SFHS

Chronic Pain was positively correlated in GS:SFHS with MDD (Pearson’s phenotypic correlation: r = 0.13, SE = 0.01, *p* = 2.72 x10^-68^). Phenotypic covariance between chronic pain and MDD was demonstrably attributable to shared genetic architecture (r = 0.51, SE = 0.054, *p* = 8.24x10^-19^) and also to a spouse/partner environmental effect (r = 0.53, SE = 0.24, *p* = 0.02). The larger standard errors (SE = 0.24) for the effect of partner/spouse likely reflected the smaller number of spouse/partners (*n* = 3,486) and the lower precision in these estimates.

### The Associations of Polygenic Risk of Pain on chronic pain in GS:SFHS and UK Biobank

**Fig 1:**
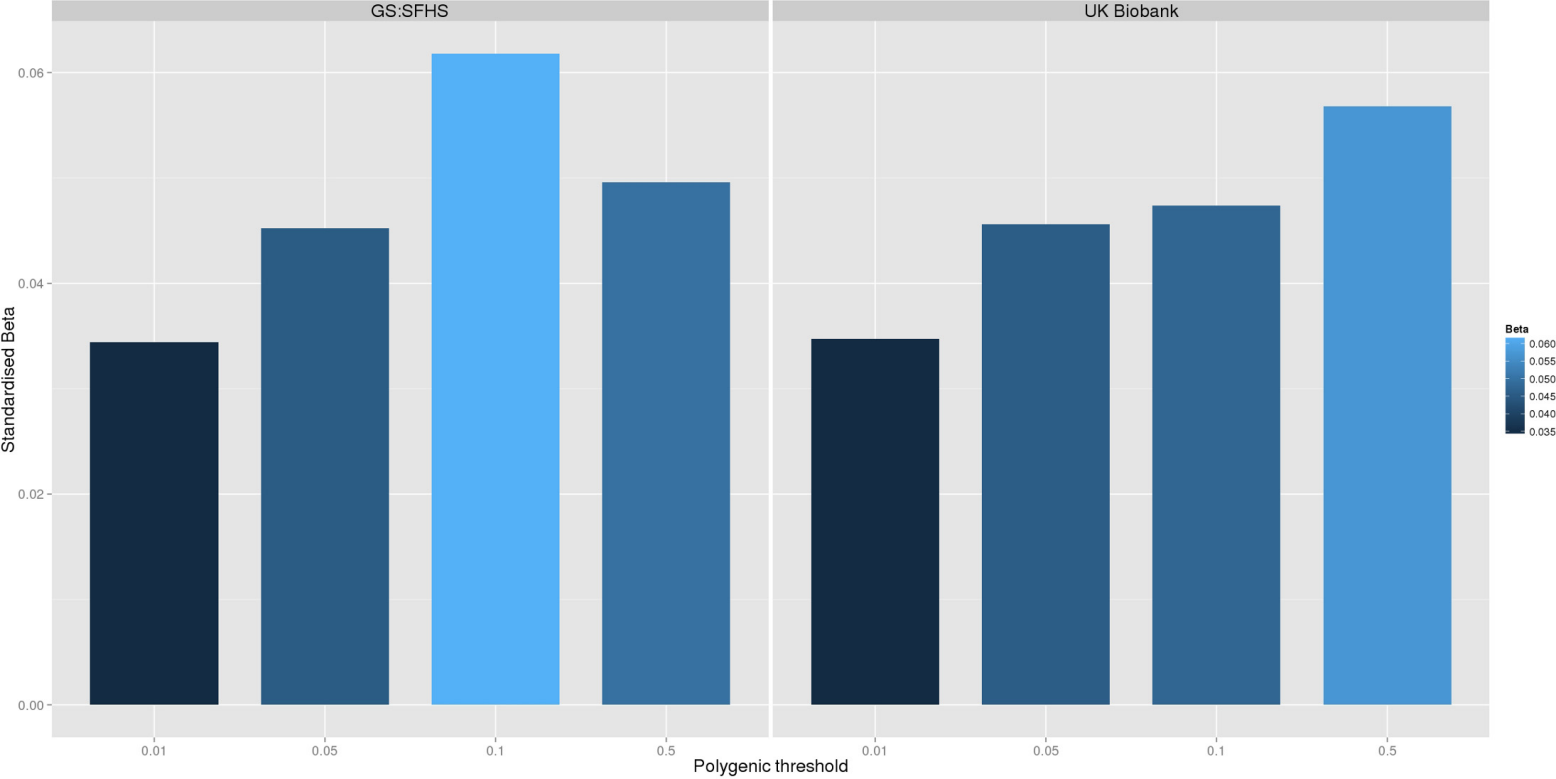
**The association between Pfizer-23andMe–derived polygenic risk profiles scores for pain on chronic pain phenotypes in GS:SFHS (left panel) and UK Biobank (right panel).** This figure shows the association between polygenic risk scores for pain (derived from Pfizer-23andMe data) and chronic pain in GS:SFHS (left panel) and UK Biobank (right panel). Vertical *y*-axis represents the effect size as a standardised beta; horizontal axis represents the four alternative *p*-value thresholds used for the generation of polygenic scores in the discovery GWAS studies.

Polygenic risk profile scores for the Pfizer-23andMe phenotype “Chronic Pain Grade” were significantly associated with chronic pain in both the GS:SFHS and UK Biobank samples, at all *p*-value thresholds (Table 1 and Fig 1). In each case, the proportion of variance in chronic pain explained by the polygenic risk score was less than 1%. In contrast, chronic pain polygenic risk profile scores derived from Pfizer-23andMe marker data were not associated with MDD in GS:SFHS at even nominal levels of significance (Table 2).

**Table 1:** The association between Pfizer-23andMe–derived polygenic profiles scores for chronic pain and chronic pain in GS:SFHS and UK Biobank.

Polygenic score	Chronic Pain Grade in Generation Scotland	Chronic Pain Grade in UK Biobank
Chronic Pain score pT = 0.01	β = 3.44 x 10^−2^ (95%CI 2.64 x 10^−3^ to 7.06 x 10^−2^), *p* = 4.20 x 10^−2^	β = 3.47 x 10^−2^ (95%CI 2.47 x 10^−2^ to 4.46 x 10^−2^), *p* ≤ 3 x 10^−4^
Chronic Pain score pT = 0.05	β = 4.51 x 10^−2^ (95%CI 1.49 x 10^−2^ to 8.32 x 10^−2^, *p* = 4.3 x 10^−3^	β = 4.65 x 10^−2^ (95%CI 3.63 x 10^−2^ to 5.62 x 10^−2^), *p* ≤ 3 x 10^−4^
Chronic Pain score pT = 0.1	β = 6.18 x 10^−2^ (95%CI 2.84 x 10^−2^ to 9.35 x 10^−2^), *p* = 4.3 x 10^−4^	β = 4.74 x 10^−2^ (95%CI 3.80 x 10^−2^ to 5.75 x 10^−2^), *p* ≤ 3 x 10^−4^
Chronic Pain score pT = 0.5	β = 4.96 x 10^−2^ (95%CI 2.11 x 10^−2^ to 8.76 x 10^−2^), *p* = 4.3 x 10^−4^	β = 5.68 x 10^−2^ (95%CI 4.70 x 10^−2^ to 6.65 x 10^−2^), *p* ≤ 3 x 10^−4^

pT represents the *p*-value threshold (in the independent discovery GWAS sample) used to calculate the polygenic risk score. β represents standardised regression coefficient between the four polygenic risk scores and the two chronic pain phenotypes. All analyses were conducted in MCMCglmm. The 95% CI represents the 95% credible interval.

**Table 2:** Association of polygenic profile scores for chronic pain and MDD in GS:SFHS.

	Association with MDD in Generation Scotland
Chronic Pain score pT = 0.01	β = 1.17x10^-2^ (95%CI -3.25 x 10^−2^ to 6.60 x 10^−2^), *p* = 0.58
Chronic Pain score pT = 0.05	β = 8.29 x 10^−3^ (95%CI -3.05 x 10^−2^ to 5.69 x 10^−2^), *p* = 0.55
Chronic Pain score pT = 0.1	β = 2.63 x 10^−2^ (95%CI -2.22 x 10^−2^ to 6.62 x 10^−2^), *p* = 0.31
Chronic Pain score pT = 0.5	β = 2.79 x 10^−2^ (95%CI -1.90 x 10^−2^ to 6.9 x 10^−2^), *p* = 0.26

MDD: Major Depressive Disorder. pT represents the *p*-value threshold (in the independent discovery GWAS sample) used to calculate the polygenic risk score. β represent the standardised regression coefficient between each pain polygenic risk score and MDD in Generation Scotland (GS:SFHS).

### The Association of Polygenic Risk for MDD with MDD Traits in GS:SFHS and UK Biobank

Polygenic risk profile scores for MDD were estimated in GS:SFHS and UK Biobank using data from the PGC international consortia. Polygenic risk profile scores for MDD were significantly positively associated with MDD in GS:SFHS at three out of four thresholds, with the exception of the pT = 0.01 threshold. In the larger UK Biobank study, polygenic risk of MDD was associated with MDD at all four *p*-value thresholds (Table 3).

**Table 3:** Association between MDD-related traits in GS:SFHS and UK Biobank with the Psychiatric Genomics Consortium derived MDD polygenic risk scores.

PGRS score	MDD in GS:SFHS	MDD in UK Biobank
MDD score pT = 0.01	β = 3.18 x 10^−2^ (95%CI -1.56 x 10^−2^ to 7.28 x 10^−2^), *p* = 0.21	β = 4.90 x 10^−2^ (SE = 1.34 x 10^−2^), *p* = 2.6 x 10^−4^
MDD score pT = 0.05	β = 7.44 x 10^−2^ (95%CI 2.71 x 10^−2^ to 1.18 x 10^−1^), *p* = 2.1 x 10^−3^	β = 7.02 x 10^−2^ (SE = 1.35 x 10^−2^), *p* = 2.1 x 10^−7^
MDD score pT = 0.1	β = 9.75 x 10^−2^ (95%CI 4.53 x 10^−2^ to 1.35 x 10^−2^), *p* ≤ 2 x 10^−4^	β = 7.38 x 10^−2^ (SE = 1.37 x 10^−2^), *p* = 6.4 x 10^−8^
MDD score pT = 0.5	β = 9.68 x 10^−2^ (95%CI 4.48 x 10^−2^ to 1.33 x 10^−1^), *p* ≤ 2 x 10^−4^	β = 6.11 x 10^−2^ (SE 1.37 x 10^−2^), *p* = 8.10 x 10^−6^

MDD: Major Depressive Disorder. pT represents the *p*-value threshold (in the independent discovery GWAS sample) used to calculate the polygenic risk score. β represents standardised regression coefficient between the four polygenic risk scores and MDD in GS:SFHS and UK Biobank. All analyses were conducted in MCMCglmm. The 95% CI represents the 95% credible interval, which is broadly interpreted as the interval in which there is a 95% probability that the true parameter lies.

Having established a relationship between polygenic risk of MDD, MDD in GS:SFHS, and probable MDD in UK Biobank, we then sought to test the relationship between polygenic risk of MDD and chronic pain. Polygenic risk of MDD was associated with chronic pain in GS:SFHS at three out of four thresholds (Table 4). The threshold of pT = 0.01 did not show a significant association with chronic pain in GS:SFHS. This was the same threshold that showed no association with MDD in the same study.

**Fig 2:**
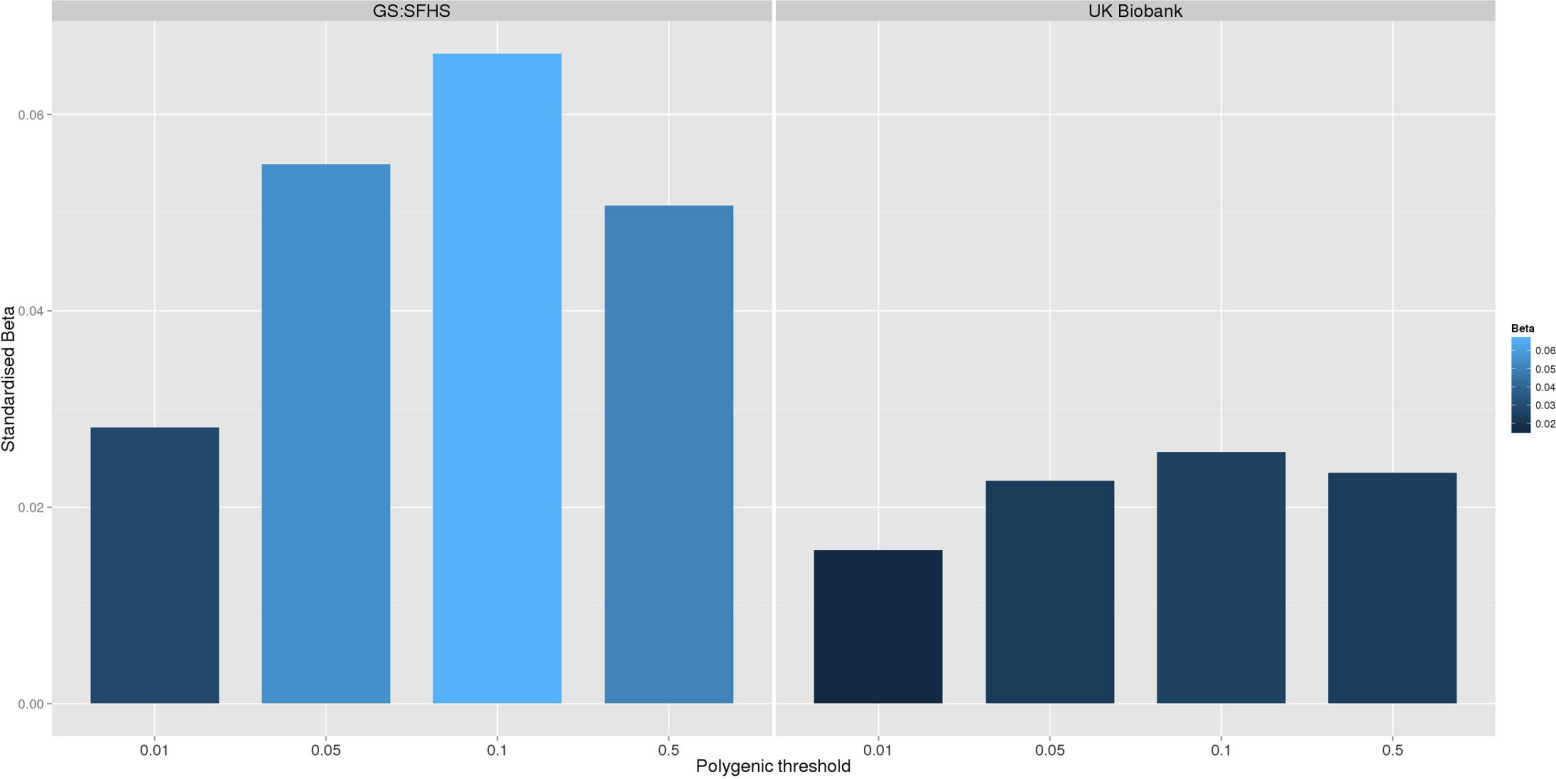
Association between polygenic risk of MDD and chronic pain phenotypes in GS:SFHS and UK Biobank. This figure shows the association between polygenic risk scores for MDD (derived from Psychiatric Genomics Consortium data) and chronic pain in GS:SFHS (left panel) and UK Biobank (right panel). Vertical *y*-axis represents the effect size as a standardised beta, horizontal axis represents the four alternative *p*-value thresholds used for the generation of polygenic scores in the discovery GWAS studies.

In order to confirm the novel association between polygenic risk of MDD and pain in GS:SFHS, we conducted a replication in the UK Biobank sample. In this sample, 121,052 UKB individuals provided phenotypic and genotypic data for analysis. Polygenic risk of MDD (at four thresholds) was positively and significantly associated with each of the pain phenotypes in UKB (Table 4 and Fig 2).

**Table 4:** Association between chronic pain in Generation Scotland and UK Biobank and polygenic scores for MDD derived using data from the Psychiatric Genomic Consortium.

Polygenic score	Chronic Pain Grade in Generation Scotland	Chronic Pain Grade in UK Biobank
MDD score pT = 0.01	β = 2.81 x 10^−2^ (95%CI -5.56 x 10^−3^ to 6.22 x 10^−2^), *p* = 9.7 x 10^−2^	β = 1.56 x10^-2^ (95%CI 6.36 x 10^−3^ to 2.60 x 10^−2^), *p* = 1.5 x 10^−3^
MDD score pT = 0.05	β = 5.49 x 10^−2^ (95%CI 2.40 x 10^−2^ to 9.16 x 10^−2^), *p* = 1.70 x 10^−3^	β = 2.27 x 10^−2^ (95%CI 1.27 x 10^−2^ to 3.21 x 10^−2^), *p* < 3 x 10^−4^
MDD score pT = 0.1	β = 6.62 x 10^−2^ (95%CI 2.82 x 10^−2^ to 9.76 x 10^−2^), *p* = 4.3 x 10^−4^	β = 2.56 x 10^−2^ 95%CI 1.62 x 10^−2^ to 3.63 x 10^−2^, *p* < 3 x 10^−4^
MDD score pT = 0.5	β = 5.07 x 10^−2^ (95%CI 1.69 x 10^−2^ to 8.35 x 10^−2^), *p* = 1.3 x 10^−3^	β = 2.35 x 10^−2^, 95%CI 1.47 x 10^−2^ to 3.37 x 10^−2^, *p* < 3 x 10^−4^

MDD: Major Depressive Disorder. pT represents the *p*-value threshold (in the independent discovery GWAS sample) used to calculate the polygenic risk score. β represents standardised regression coefficient between the four polygenic risk scores and the two chronic pain phenotypes. All analyses were conducted in MCMCglmm. The 95% CI represents the 95% credible interval, which is broadly interpreted as the interval in which there is a 95% probability that the true parameter lies.

## Discussion

In GS:SFHS, genetic factors and shared spouse/partner environment contributed significantly to variation in chronic pain. Narrow sense heritability of chronic pain was 38.4%, the effect of spouse additionally contributed 18.7% to phenotypic variance, and 42.9% of phenotypic variance was left to residual error and other unknown sources of variation. Chronic pain was also positively correlated with MDD, and this correlation was due to both shared genetic factors and to an effect of spouse/partner.

Using data from two independent GWAS studies (Pfizer-23andMe and PGC), polygenic profile scores for chronic pain were associated with chronic pain traits in both GS:SFHS and UK Biobank, but not MDD. In contrast, polygenic risk of MDD was positively and significantly associated with MDD and chronic pain in both GS:SFHS and UK Biobank. In a study with some similarities to ours, Ligthart et al. [38] calculated genetic risk scores, based on two discovery samples, for migraine and for MDD, and were able to predict “pure” migraine cases and “comorbid” (migraine and MDD) cases in an independent Dutch sample. This study extends that earlier work to a broader chronic pain phenotype, in independent samples, using complimentary and convergent methodologies.

This investigation replicates and extends earlier work showing that chronic pain is a heritable phenotype [39] by demonstrating that there is an independent contribution to risk from the environment shared by an individual’s partner or spouse. We also demonstrate that chronic pain has a partly polygenic risk architecture, further supporting analyses aimed at identifying specific risk-associated variants. Whilst pain is phenotypically and genetically correlated with MDD, the added contribution of an affected spouse was also substantial in the current study. The reasons for this are unclear in the current study, but the findings imply that the presence of pain in one spouse/partner makes the probability of MDD more likely in the other spouse/partner. Possible reasons for this include learned behaviours and the effects of caring for someone with chronic disabling illness. An alternative explanation is that a spousal correlation represents the impact of a common recent environment jointly on both spouses. Spouses are a group who are presumed to have been living together, on the basis of shared children and co-participation in the study. Their correlation could represent common infectious disease history, diet, deprivation, hobbies, and a number of as yet unidentified shared factors. In addition, an effect of assortative mating cannot be excluded.

Whilst tests of genetic correlation imply shared genetic risk factors, they cannot ascertain the mechanisms of any effect. Similarly, they cannot determine whether the presence of chronic pain in one individual is associated with a risk of depression in another because of shared mechanisms or because one phenotype is the consequence of the other. Interestingly, in our analyses, chronic pain was associated with increased genomic risk for MDD. There was, however, no significant association of MDD with genomic risk of pain after correction for multiple testing. These results, whilst they potentially imply a directional association, may, however, be due to subtle differences in genetic architectures, discovery GWAS sample sizes, GWAS quality-control, or polygenic risk profiling. It is also possible that a higher genetic risk of MDD lowers the threshold at which pain is perceived or reported, even if an individual does not develop frank psychiatric disorder. The finding of a robust MDD polygenic risk score association with pain is unexpected, as MDD has (to date) not been particularly tractable to genetic studies. This finding implies that there is a potentially important genetic relationship between MDD and pain.

Several limitations should be considered when interpreting the findings from this study. Firstly, response rates to a letter of initial invitation in population-based studies are typically low (around 12.3% for GS:SFHS [24], 5.5% for UK Biobank [40], and 13.6% for 23andMe). This may decrease the representativeness of each cohort and could, in theory, bias the effect size estimates [40]. The Pfizer-23andMe survey was also deployed electronically via email and on the 23andMe website to the entire 23andMe research participant cohort. Whilst the effects of this method of participant selection on genetic studies is not known, the ability of Pfizer-23andMe polygenic pain scores to predict pain scores in two independent cohorts provides some assurance of consistency with other studies.

Whilst GS:SFHS is a large family-based study, the genetic effect estimated in the pedigree-based study tends to be inflated by shared environmental factors. Whilst we applied step-wise model selection to adjust for the effects of shared environment, the effects of environment factors with small effects could not be completely excluded. Whilst the use of the DIC criterion for non-Gaussian models is subject to several important limitations [41], the lower DIC values for the models including spouse only are less dependent on sample size and provide additional convergent information to the comparison of log-likelihoods. A separate issue to take account of is the fact that the utility of polygenic risk scores depends largely on the precision of the marker estimates in the independent discovery set. The precision of these estimates from Pfizer-23andMe and the PGC are dependent upon a number of factors, including sample size, allele frequency, and heritability. The lack of an association with polygenic risk of chronic pain and MDD may be due in part to poorly estimated marker effect sizes or to unidentified factors related to differences in the cohorts. Excluding this possibility may require much larger discovery GWAS sample sizes. However, in contrast to complex phenotypes of comparable heritability, GWAS studies of MDD in individuals of European ancestry have so far yielded no significant findings. It is perhaps surprising, therefore, that risk of MDD should be significantly associated with chronic pain in both GS:SFHS and UK Biobank. Whilst this finding could be due to pleiotropy, or a directional effect of polygenic risk of MDD on pain, there is a possibility that some cases of MDD are misclassified as chronic pain, or vice versa, leading to inflated estimates of genetic correlation and polygenic association with MDD. Such a misclassification could occur for a number of reasons, including the voicing or manifestation of distress through physical rather than psychological language.

Arguably, one potential limitation to the current study is the use of different definitions of chronic pain and MDD in GS:SFHS and UKB. In spite of this difference, the presence of an association between polygenic risk of MDD and chronic pain in both cohorts further highlights the strength and robustness of the pain-MDD relationship. The findings also signpost the availability of three suitable cohorts (GS:SFHS, UKB and Pfizer-23andMe) of sufficient size and power in which to conduct molecular genetic studies to better understand the shared mechanisms of chronic pain and MDD.

The findings from the current investigation imply that efforts to identify genetic and environmental risk factors for MDD are likely to be relevant for the study of pain, and vice versa. We think that future efforts in this field should include a search to identify the risk-conferring loci contributing to chronic pain and MDD and, thereafter, a search to identify the direction of the causal mechanisms that link these disorders. The answer to these key questions are likely to signpost new directions for therapeutic interventions and highlight the symptoms that are most amenable to treatment, as well as to prevention.

In conclusion, we show here that shared genetic factors and recent shared environment with a spouse/partner contribute to variation in chronic pain and also to its observed co-occurrence with MDD. Our conclusions are made based upon bivariate mixed-model analysis of a family-based study and on convergent and replicated evidence using polygenic risk scores from two independent studies.

## Supporting Information

S1 Table Best fitting pedigree models for chronic pain in the GS:SFHS cohort. (DOCX)

S2 Table Table of SNPs included in polygenic risk score calculations. (DOCX)

S1 Text Construction of environmental variables in GS:SFHS. (DOCX)

S2 Text Details of genotyping and polygenic profiling. (DOCX)

## Abbreviations

CPG: Chronic Pain Grade
CWP: chronic widespread pain
DIC: Deviance Information Criterion
DSM: Diagnostic and Statistical Manual of the American Psychiatric Association
GS:SFHS: Generation Scotland: Scottish Family Health Study
GWAS: genome-wide association studies
MDD: major depressive disorder
PGC: Psychiatric Genomics Consortium
PGRS: polygenic risk score
SCID: Structured Clinical Interview
SNP: single nucleotide polymorphism
UKB: UK Biobank
