# Supplementary material for: Genetic and Environmental Risk for Chronic Pain and the Contribution of Risk Variants for Major Depressive Disorder: A Family-Based Mixed-Model Analysis
Source: PLoS Med. 2016 Aug 16;13(8):e1002090. doi: 10.1371/journal.pmed.1002090 (PMC4987025; doi:10.1371/journal.pmed.1002090)
Supplement: S1 Table — (DOCX) [file pmed.1002090.s001.docx]

**Supplementary Table 1: Best fitting models for chronic pain in GS:SFHS**

|  | MCMCglmm Test | ASReml-R LogLik test | Effect sizes (95%CI) |
| --- | --- | --- | --- |
| Additive only (A) | DIC = 31176 | - | A = 35.54% (31.91% to 41.78%) |
| A + sib | DIC = 31087 | χ^2^ = 4.58, p = 0.03 | A = 37.04% (31.37% to 42.93%)  Sib = 0.06% (7.26x10^-3^% to 2.73%) |
| A + spouse | DIC = 28787 | χ^2^ = 27.23, p = 1.80 x10^-7^ | A = 38.29% (33.06% to 44.03%)  Spouse = 18.66% (10.83% to 25.06%) |
| A + household | DIC = 30885 | χ^2^ = -4.06, p = 0.13 | A = 32.23% (26.17% to 40.00%)  Household = 5.65% (2.08% to 11.58%) |
| A + spouse + sib | DIC = 28956 | χ^2^ = 0.50, p = 0.48 | A = 37.11% (32.03% to 42.28%)  Spouse = 15.83% (9.30% to 23.15%)  Sib = 0.20% (0.11% to 2.70%) |
| A + spouse + household | DIC = 28902 | χ^2^ = -3.17 x10^-5^, p = 1.0 | A = 38.52% (31.75% to 44.76%)  Spouse = 21.15% (8.81% to 25.24%)  Household = 0.09% (0.04% to 6.57%) |

DIC: Deviance Information Criterion. Household represents the designated effects from the ‘old household’ and ‘young household
