# Supplementary material for: Genetic and Environmental Risk for Chronic Pain and the Contribution of Risk Variants for Major Depressive Disorder: A Family-Based Mixed-Model Analysis
Source: PLoS Med. 2016 Aug 16;13(8):e1002090. doi: 10.1371/journal.pmed.1002090 (PMC4987025; doi:10.1371/journal.pmed.1002090)
Supplement: S2 Table — (DOCX) [file pmed.1002090.s002.docx]

**S2 Table: Table of SNPs included in polygenic risk score calculations**

|  |  |  |  |  |  |
| --- | --- | --- | --- | --- | --- |
|  |  | P = 0.01 | P = 0.05 | P= 0.1 | P = 0.5 |
| GS:SFHS | MDD | 2853 | 10855 | 18970 | 63112 |
| GS:SFHS | Pain | 3012 | 12169 | 21177 | 68979 |
| UK Biobank | MDD | 1532 | 6878 | 12995 | 56175 |
| UK Biobank | Pain | 3464 | 15720 | 29705 | 126337 |
