# Supplementary material for: Genetic and Environmental Risk for Chronic Pain and the Contribution of Risk Variants for Major Depressive Disorder: A Family-Based Mixed-Model Analysis
Source: PLoS Med. 2016 Aug 16;13(8):e1002090. doi: 10.1371/journal.pmed.1002090 (PMC4987025; doi:10.1371/journal.pmed.1002090)

**Construction of environmental variables in GS:SFHS**

These included an estimate of the effect of common parents, whereby individuals who shared two parents were assigned a same unique number in a ‘full-sibling’ variable, which reflects the shared environment of full-siblingss. Secondly, we estimated the shared environmental effect between couples by assigning the same unique number to each pair of individuals (with different sex) with a common child in a variable ‘spouse’. Thirdly, we created two household variables: ‘old-household’ and ‘young-household’. The household variables were used in order to distinguish the household effect which only involves nuclear family members from the genetic effect tracked by both close and distant relatives. The household effect is reflected by assigning a same unique number for all members who live in the same household. For large families with a three-generation family structure (the family contains grandparents, parents and grandchildren), the design of the two variables corresponded to the shared household environment of the older-nuclear family made up of grand-parents and parents, and the younger-nuclear family made up of parents and grandchildren. For small families with a two generation structure (the family only contains parents and children), the household effect from the nuclear family will be reflected in the old-household variable if the average age of the parents is larger than 59, whereas the household effect will be reflected in the young-generation variable if the average age of the parents was less than 59. A procedure for selecting appropriate age cut-off was derived as follows: First, families with a three-generation structure and families with a two-generation structure were identified. Among families with a three-generation structure, the mean age of the parents in older-nuclear-families was 74 and the mean age of the parents in younger-nuclear-families was 49. Secondly, the age cut-off of 59 for the families with two-generation structure was selected by testing the empirical age cut-offs from 49 to 74 (step=1). The cut-off that minimized the sum of the variance of age between the nuclear families included in the old-household variable and the variance of age between the nuclear families included in the young-household variable was selected and found to be 59. This cut-off of 59 years also produced approximately equal numbers in the old-nuclear family group and the young-nuclear family group.

**Supplementary material: derivation of sib, spouse and household variables**


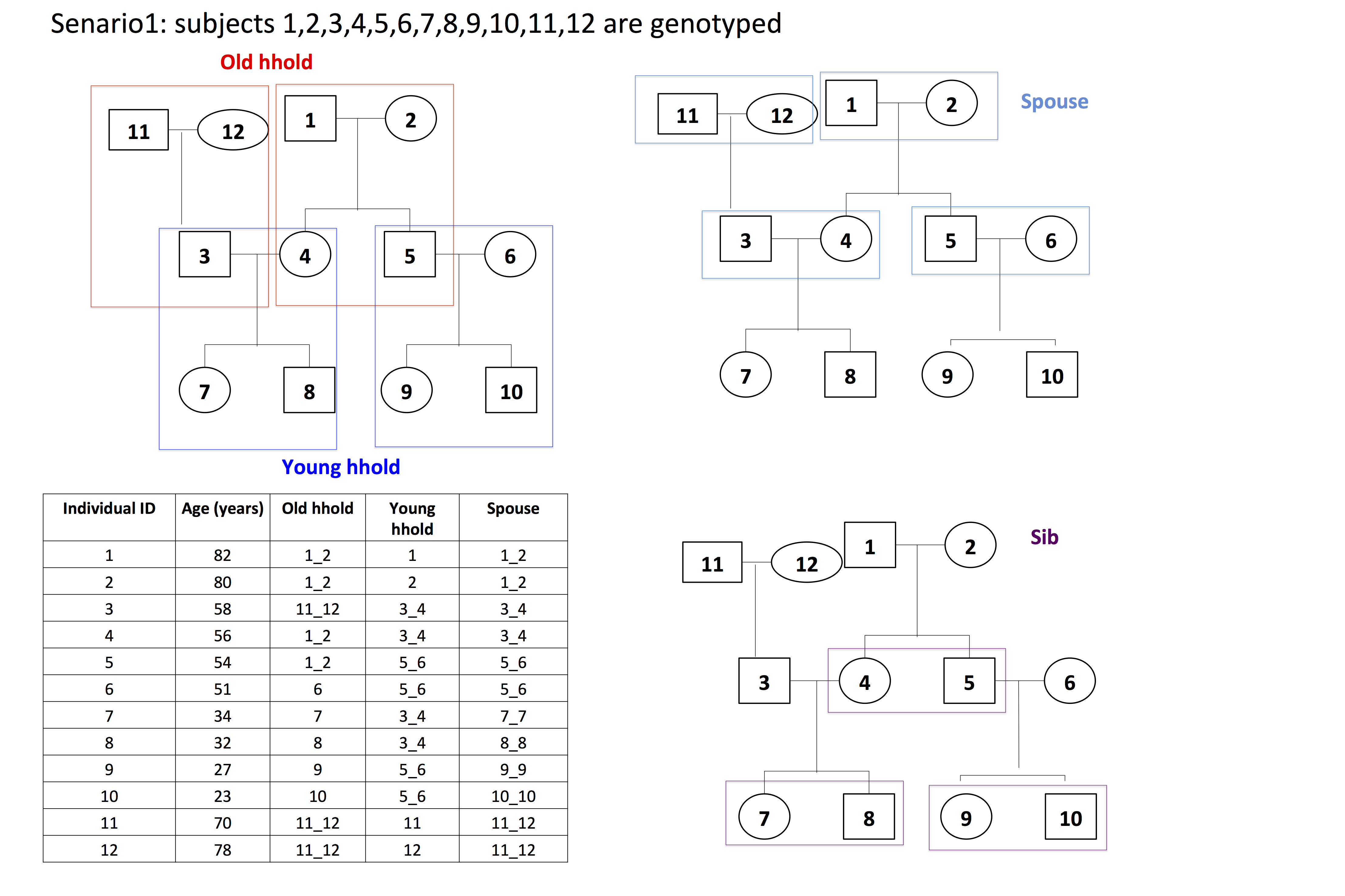


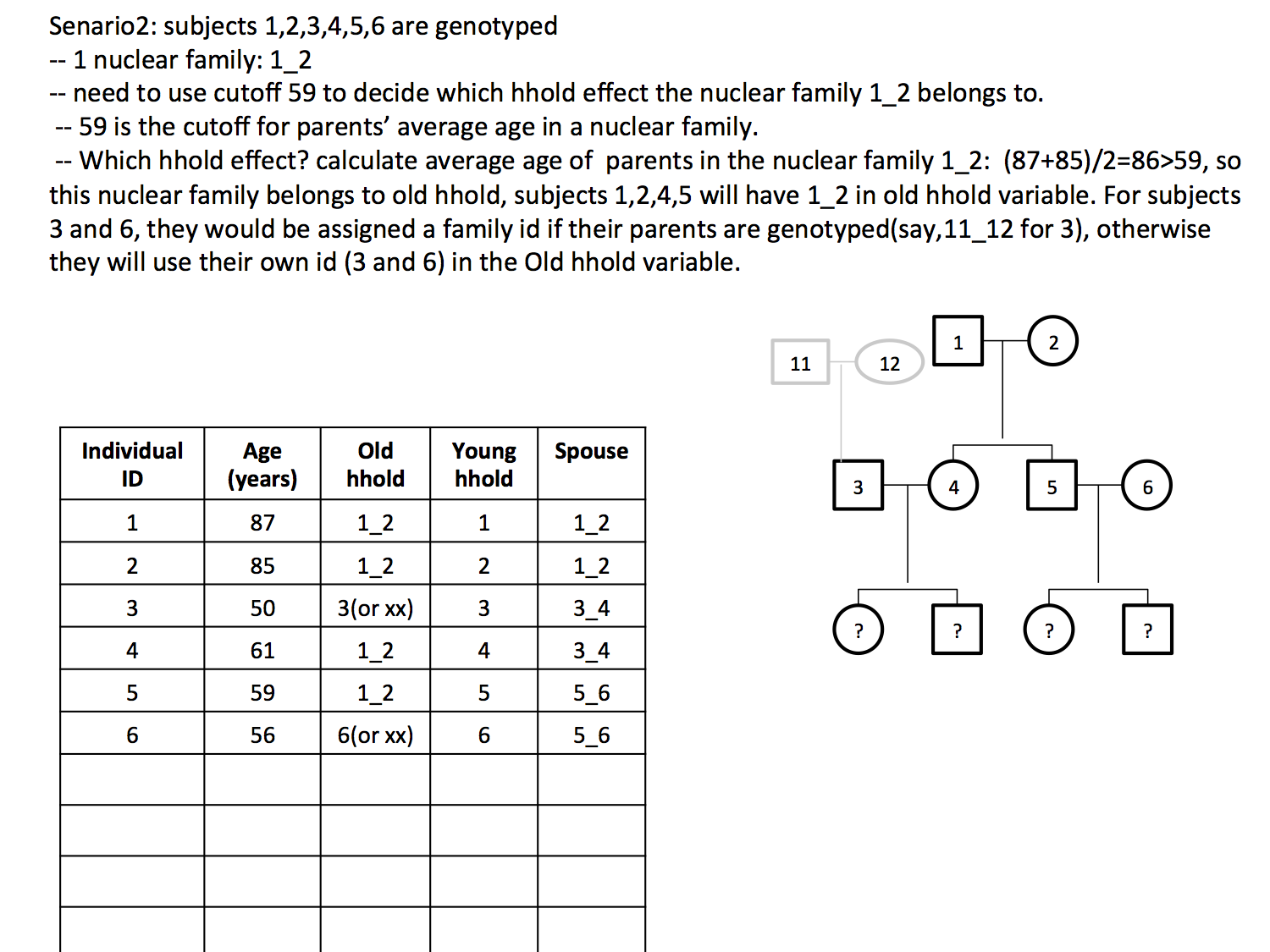


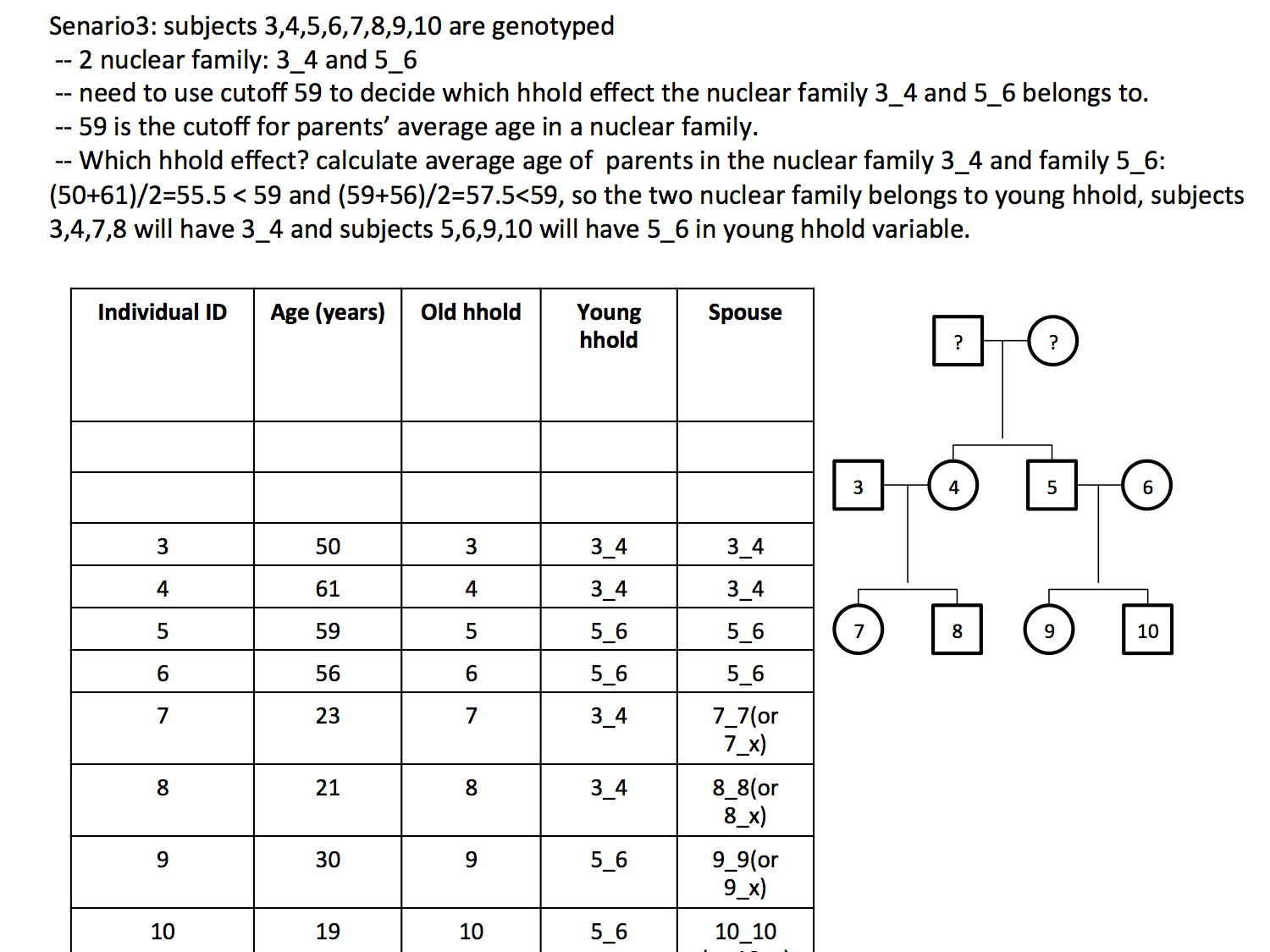


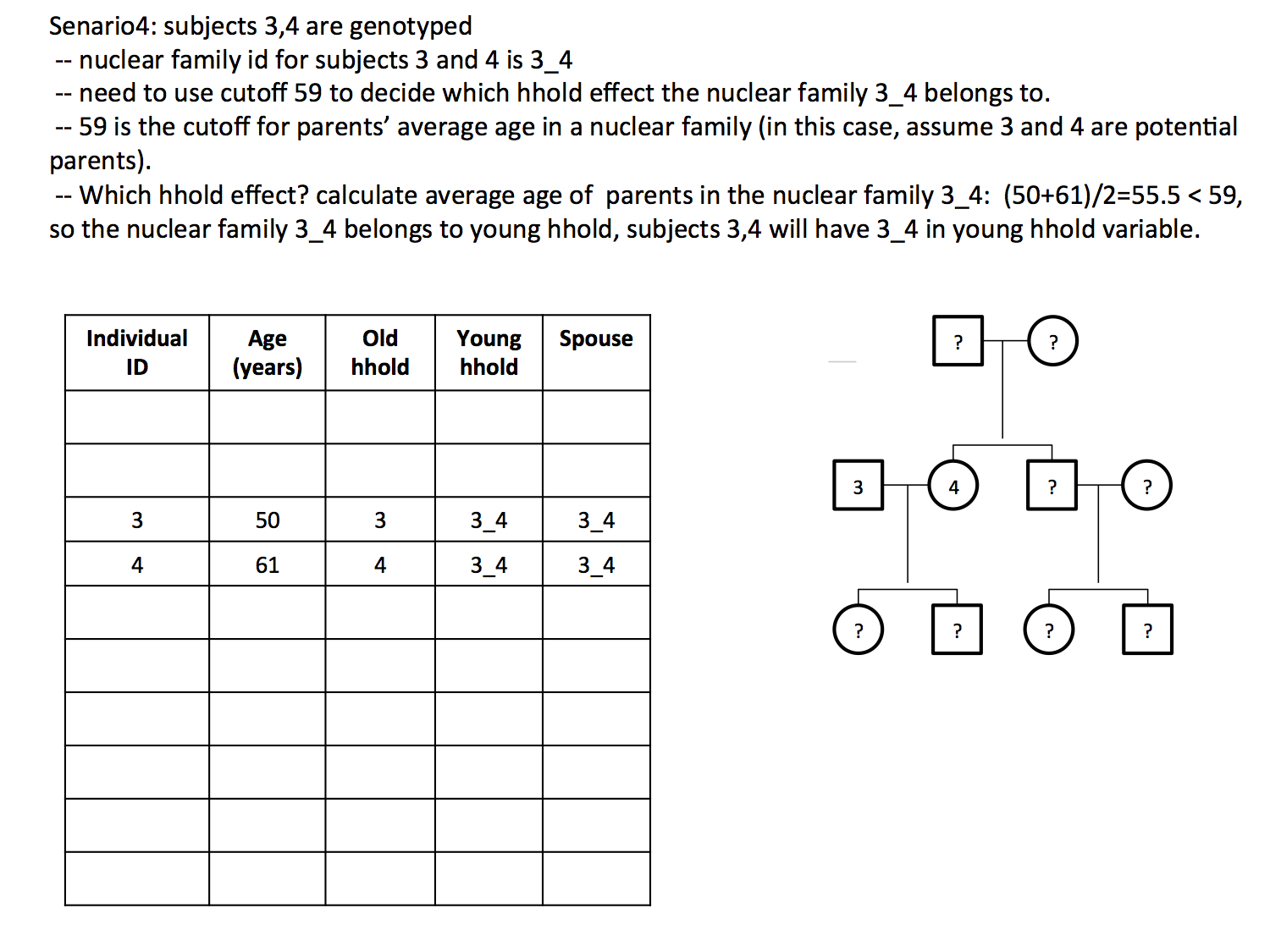


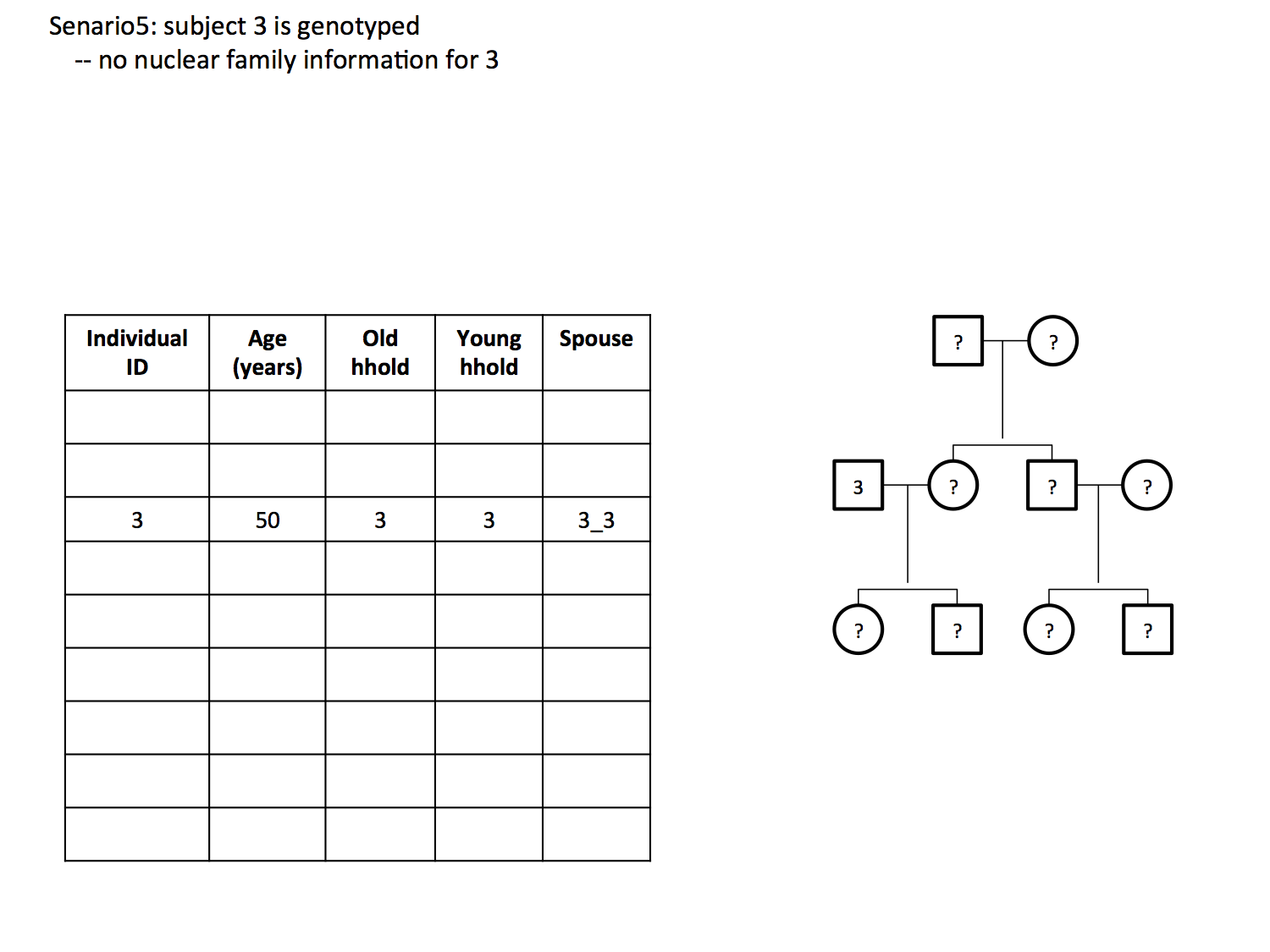

Supplement: S1 Text — (DOCX) [file pmed.1002090.s003.docx]
