# Supplementary material for: Genetic and Environmental Risk for Chronic Pain and the Contribution of Risk Variants for Major Depressive Disorder: A Family-Based Mixed-Model Analysis
Source: PLoS Med. 2016 Aug 16;13(8):e1002090. doi: 10.1371/journal.pmed.1002090 (PMC4987025; doi:10.1371/journal.pmed.1002090)
Supplement: S2 Text — (DOCX) [file pmed.1002090.s004.docx]

**Genotyping in GS:SFHS**

Individuals were genotyped using the Illumina HumanOmniExpressExome-8v1.0 BeadChip and Infinum chemistry24 and processed using the IlluminaGenomeStudio Analysis software v2011.1 (Illumina, San Diego, CA, USA).

Quality control on raw genotypes removed individuals with an overall genotyping rate of <99%, SNPs with a minor allele frequency <1%, call rate <99% or a significant deviation from Hardy–Weinberg equilibrium (P < 1 × 10^−6^). Ancestry informative principal components were created in GCTA to identify outliers for later removal. Four GS:SFHS population outliers were removed and four multidimensional scaling components were used to correct for population stratification in the remaining individuals.

**Genotyping in UK Biobank**

152 729 UK Biobank blood samples were genotyped using either the UK BiLEVE array (N = 49 979) or the UK Biobank axiom array (N = 102 750). Genotyping was performed in batches of approximately 4700 samples by Affymetrix, who also performed quality control of the genotyping data. Further details are available of the genotyping and quality control (QC) procedures are available online (http://biobank.ctsu.ox.ac.uk/crystal/refer.cgi?id=155583) including the Axiom array (http://media.affymetrix.com/support/downloads/manuals/axiom_2_assay_auto_workflow_user_guide.pdf). A further QC protocol was then applied (<http://biobank.ctsu.ox.ac.uk/crystal/refer.cgi?id=155580)> to the genotype data at the Wellcome Trust Centre for Human Genetics (WTCHG) before the data was released to the study team.

Prior to further analyses individuals were removed based on missingness, relatedness (KING estimated kinship co-efficient > 0.0442), gender mismatch, non-British ancestry (principal component analysis identified probable Caucasians within those individuals that were self-identified as British), and QC failure in the UK Bileve study. A sample of 112 151 individuals remained for further analyses. A minor allele frequency of maf < 1% filter was applied and only autosomal variants were used (N = 705 516).

**Genotyping in Pfizer-23andMe sample**

DNA from saliva samples was genotyped on one of two platforms. About 35% of the participants were genotyped on the Illumina HumanHap550+ BeadChip platform, which included SNPs from the standard HumanHap550 panel augmented with a custom set of approximately 25,000 SNPs selected by 23andMe. The remaining 65% of participants were genotyped on the Illumina HumanOmniExpress+ BeadChip. This platform has a base set of 730,000 SNPs augmented with approximately 250,000 SNPs to obtain a superset of the HumanHap550+ content, as well as a custom set of about 30,000 SNPs. Individuals included in the analysis were selected for having >97% European ancestry, as determined through an analysis of local ancestry via comparison to the CEU, YRI and JPT + CHB HapMap 2 populations. A maximal set of unrelated individuals was chosen for the analysis using a segmental identity-by-descent (IBD) estimation algorithm. Individuals were defined as related if they shared more than 700 cM of IBD, including regions where the two individuals shared either one or both genomic segments IBD. This level of relatedness (involving roughly 20% of the genome) corresponds approximately to the minimal expected sharing between first cousins in an outbred population.

Participant genotype data were imputed against the August 2010 release of the 1000 Genomes Project reference haplotypes. BEAGLE (version 3.3.1) was used to phase batches of 8,000 to 9,000 individuals across chromosomal segments of no more than 10,000 genotyped SNPs, with overlaps of 200 SNPs. The following were excluded: SNPs with minor allele frequency (MAF) < 0.001, Hardy-Weinberg equilibrium P < 1 × 10^-20^, call rate < 95% or large allele frequency discrepancies compared to the 1000 Genomes Project reference data. Fully phased chromosomes were assembled by matching the phase of haplotypes across the overlapping segments. Each batch was imputed against the European subset of 1000 Genomes Project haplotypes using Minimac (2011-10-27) with 5 rounds and 200 states for parameter estimation. Analyses were limited to 7.4 million SNPs with imputed r^2^ > 0.5 averaged across all batches and r^2^ > 0.3 in every batch.

**Polygenic profiling**

Polygenic profiling of GS:SFHS and UK Biobank was conducted according to previous published work in the PRSice software package[1].

In GS:SFHS, directly genotyped samples (unimputed data) was used for the calculation of polygenic risk profile scores using either the Psychiatric Genomics Consortium Major Depressive Disorder Working Group data (PGC1-MDD)[2] or the summary GWAS results from Pfizer-23andMe. SNP data with a minor allele frequency of 1% or more was used using an r^2^ threshold of 0.1 over a 250Kb sliding window.

Polygenic risk profile scores for MDD were also calculated in UK Biobank for a previous study, employing the same (unimputed) GWAS data, using a 1% minor allele frequency and a r^2^ threshold of 0.25 and a sliding window of 200Kb. Pain polygenic risk scores were calculated later using the Pfizer-23andMe marker weights and the imputed GWAS data, also using a 1% minor allele frequency, an r^2^ threshold of 0.25 and a sliding window of 200Kb.

1. Euesden J, Lewis CM, O'Reilly PF. PRSice: Polygenic Risk Score software. Bioinformatics. 2015;31(9):1466-8. doi: 10.1093/bioinformatics/btu848. PubMed PMID: 25550326; PubMed Central PMCID: PMCPMC4410663.

2. Major Depressive Disorder Working Group of the Psychiatric GC, Ripke S, Wray NR, Lewis CM, Hamilton SP, Weissman MM, et al. A mega-analysis of genome-wide association studies for major depressive disorder. Molecular psychiatry. 2013;18(4):497-511. doi: 10.1038/mp.2012.21.
